# High-Performance Nanoscale Metallic Multilayer Composites: Techniques, Mechanical Properties and Applications

**DOI:** 10.3390/ma17092124

**Published:** 2024-04-30

**Authors:** Mahmoud Ebrahimi, Bangcai Luo, Qudong Wang, Shokouh Attarilar

**Affiliations:** 1 Department of Mechanical Engineering, Faculty of Engineering, University of Maragheh, Maragheh 83111-55181, Iran; ebrahimi@maragheh.ac.ir; 2 Ningbo Major Draft Beer Equipment Co., Ltd., Ningbo 315033, China; boyce@chinesebrass.com; 3 National Engineering Research Center of Light Alloy Net Forming and Key State Laboratory of Metal Matrix Composites, School of Material Science and Engineering, Shanghai Jiao Tong University, Shanghai 200240, China; 4 Department of Materials Engineering, Faculty of Engineering, University of Maragheh, Maragheh 83111-55181, Iran; sh.attarilar@yahoo.com

**Keywords:** metallic multilayer composites, synthesis methods, tensile behavior, fatigue endurance, electrical resistivity

## Abstract

Due to their exceptional properties and diverse applications, including to magnetic devices, thermoelectric materials, catalysis, biomedicine, and energy storage, nanoscale metallic multilayer composites (NMMCs) have recently attracted great attention. The alternating layers of two or more metals that make up NMMCs are each just a few nanometers thick. The difficulties in producing and synthesizing new materials can be overcome by using nanoscale multilayer architectures. By adjusting the layer thickness, composition, and interface structure, the mechanical properties of these materials can be controlled. In addition, NMMCs exhibit unusually high strength at thin layer thicknesses because the multilayers have exceptionally high strength, as the individual layer thicknesses are reduced to the nanoscale. The properties of NMMCs depend on the individual layers. This means that the properties can be tuned by varying the layer thickness, composition, and interface structure. Therefore, this review article aims to provide a comprehensive overview of the mechanical properties and the application of high-performance NMMCs. The paper briefly discusses the fabrication methods used to produce these composites and highlights their potential in various fields, such as electronics, energy storage, aerospace, and biomedical engineering. Furthermore, the electrical conductivity, mechanical properties, and thermal stability of the above composite materials are analyzed in detail. The review concludes with a discussion of the future prospects and challenges associated with the development of NMMCs.

## 1. Introduction

**Figure 1:**
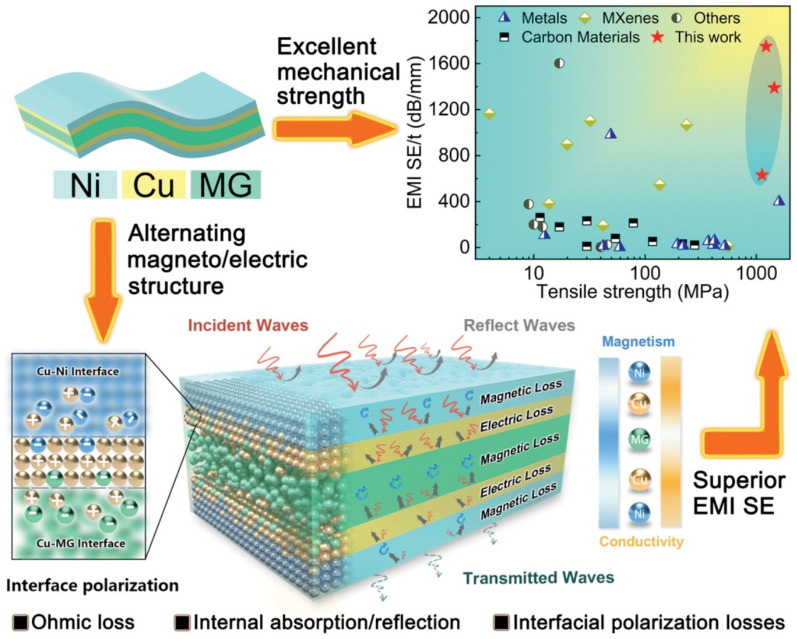
The structure of the Ni/Cu/MG multilayer composite, the electromagnetic interference (EMI) shielding effectiveness mechanism, and its mechanical performance by EMI SE/t versus tensile strength graph [8].

High-performance nanoscale metallic multilayer composites (NMMCs) have drawn a lot of interest in recent years because of their unique combination of properties and potential applications in various fields. These composites consist of alternating layers of different metallic materials, typically with layers between a few nanometers and a few micrometers thick. The precise control over layer thickness and composition allows for tailoring the properties of these composites, making them highly versatile and desirable for numerous applications, such as magnetic and electronic devices, structural materials, thermoelectric materials, catalysis, biomedicine, and energy storage. The electrical, mechanical, and thermal properties of high-performance NMMCs make them particularly attractive for use in advanced electronic devices, structural materials, and thermal management systems [1]. The electrical conductivity of these composites can be significantly enhanced, compared to their individual constituent materials, making them suitable for applications requiring efficient current flow [2,3]. Moreover, their exceptional mechanical strength and toughness make them ideal candidates for structural components that require both high strength and ductility [4,5]. In addition to their electrical and mechanical properties, the thermal conductivity of high-performance NMMCs can be tailored to meet specific requirements [6,7]. By carefully selecting the constituent materials and layer thicknesses, it is possible to achieve either high or low thermal conductivity values. This property makes these composites highly desirable for applications such as heat sinks, thermoelectric devices, and thermal barrier coatings. One investigation by Ma et al. [8] created and studied an ultrathin and flexible Ni/Cu/metallic glass/Cu/Ni (Ni/Cu/MG) multilayer composite with alternate magnetic and electrical structures. This composite was created by facial electroless plating Cu and Ni on a Fe-based metallic glass, as shown in Figure 1, showing the structure of the Ni/Cu/MG multilayer composite, its electromagnetic interference shielding effectiveness (EMI SE) mechanism, and its mechanical performance by EMI SE/t versus tensile strength graph. It should be noted that facial electroless plating is one type of metal plating that does not need an external power source. The part is cleaned with chemical cleansers and submerged in an aqueous solution, before anti-oxidant chemicals are added. Through this process, the object’s entire surface can be consistently bathed in metal ions, producing a plated part with a high level of corrosion and friction resistance [9].

**Figure 2:**
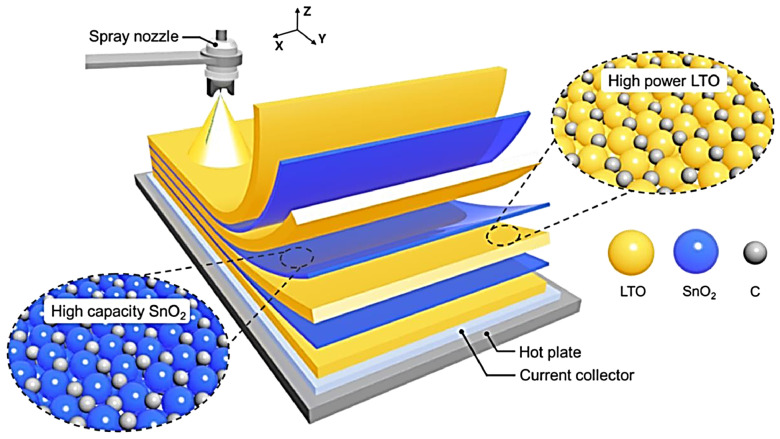
The structure of a hetero-electrode composite with discrete layers of high-power (LTO) and high-capacity SnO_2_ [10].

Ni/Cu/MG demonstrates excellent flexibility, superior tensile strength, and bending stability, along with enhanced Joule heating characteristics and thermal stability. Specifically, the Ni/Cu/MG composite has exceptional mechanical stability and a high tensile strength of up to 1.2 GPa, allowing the EMI SE to remain unchanged after 10,000 bends. The significant ohmic losses, the enhanced internal reflection/absorption, and the significant interfacial polarization loss are the causes of the promoted EMI SE [8]. In another study, Lee et al. [10] analyzed the production of multilayered composite electrodes with smart lithium-ion storage applications produced through layer-by-layer spray printing. In this regard, Figure 2 shows the structure of a layer-by-layer spray-printed hetero-electrode multilayered composite consisting of high-power Li_4_Ti_5_O_12_ (LTO) and high-capacity SnO_2_. In general, high-performance nanoscale metallic multilayer composites possess a variety of exceptional properties that differ from those observed in monolithic films, including high strength, ductility, energy absorption, thermal stability, and the enabling of self-propagating reactions [11]. The abundance of interfaces and the thickness of the layering at the nanoscale are the main factors contributing to these characteristics.

The potential applications of high-performance NMMCs are vast and diverse, including magnetic and electronic devices, structural materials, thermoelectric materials, catalysis, biomedicine, and energy storage. They have been extensively investigated for use in electronic devices, such as interconnects, electrodes, sensors, and energy storage systems [12,13]. Additionally, they hold promise in structural applications where lightweight materials with exceptional strength are required [1]. Furthermore, their tunable thermal conductivity makes them suitable for use in advanced cooling systems or as thermoelectric materials for energy conversion [14,15]. Understanding the fundamental principles governing the electrical, mechanical, and thermal behavior of high-performance NMMCs is crucial for optimizing their performance in specific applications. This paper will delve into the underlying mechanisms that contribute to their unique properties by discussing topics such as interface effects, grain boundary diffusion phenomena, dislocation interactions across layers, and size effects at the nanoscale [16].

Overall, this paper aims to provide a comprehensive overview of the applications, as well as the mechanical properties of high-performance NMMCs. The fabrication techniques used to produce these composites with precise control over layer thicknesses and compositions are discussed. Furthermore, the various characterization methods employed to evaluate their properties at the nanoscale level are presented. In conclusion, high-performance NMMCs offer exciting opportunities for advancements in various fields due to their exceptional combination of electrical conductivity enhancement and mechanical property improvement, while maintaining ductility characteristics along with tunable thermal conductivity values. This paper aims to provide a comprehensive understanding of these materials by exploring their fabrication techniques, discussing their applications across different industries, and highlighting key factors that influence their behavior, along with mechanical strength enhancement mechanisms at interfaces between layers within composite structures.

### 1.1. Background

High-performance NMMCs are a class of advanced materials that exhibit exceptional mechanical, electrical, and thermal properties. The concept of metallic multilayer composites dates back to the 1960s, when researchers first discovered that alternating layers of different metals could enhance the strength and hardness of materials [17]. These composites have very high strength due to the Orowan strengthening of the fine-scale layers [18]. However, it was not until recent advancements in nanotechnology and thin film deposition techniques that high-performance NMMCs gained significant attention [19].

Multilayer metal composites have excellent properties, such as wear resistance and synergistic strengthening and toughening behavior [20,21]. With a high fracture elongation of 24%, these composites’ ultimate tensile strength can reach 1380 MPa [20]. The unique properties exhibited by high-performance NMMCs are primarily attributed to the interface between the different metallic layers. At these interfaces, a phenomenon known as the Hall-Petch effect occurs, which leads to grain refinement and increased strength. Additionally, the presence of interfaces can hinder dislocation movement, resulting in improved mechanical properties, such as high strength, hardness, and wear resistance [22]. For instance, dislocation glide in the layers is clearly visible in the deformation microstructure of Cu/Nb multilayer composites, which reduces dislocation stacking [22].

Another important characteristic of multilayer composites is the heterostructure strengthening effect. In multilayer composites, heterostructure strengthening describes the improvement of the composite material’s mechanical characteristics as a result of interactions between various layers or structural elements. Many heterostructure types, including graphene-MoS_2_ heterostructures [23] and multilayer heterostructured composite material systems for protective armors [24], have this strengthening behavior. Analytical formulae, which take into account the stacking sequence, the number of layers (n), and whether the material is monoplanar or multiplanar, can be used to compute the effective mechanical properties of multilayer nano-heterostructures [23]. Also, for both single and multiple layers, the effective elastic moduli and Poisson’s ratios can be predicted, offering information about the mechanical properties of the composite material [23]. Because of the interactions between various phases or components within the structure, heterostructured materials can also have superior properties. Heterostructured lamellar structures have demonstrated efficacy in generating strain hardening and strengthening due to high-density indentation (HDI) [25]. The layered structure of maraging steel-carbon steel composites leads to the formation of a strain gradient and back stress-strengthening effects, which are responsible for the synergetic heterostructure effect [26].

The coordinated deformation concept in multilayer composites is a method to enhance strength and ductility by coordinating the deformation mechanisms of the different layers within the composite material [20,27,28]. The goal of this approach is to simultaneously improve the material’s ductility and strength. Multilayer graphene (MLG) in graphene/aluminum composites is one example of this, as it has been demonstrated to enhance the composite material’s strength, ductility, and fracture behavior through a coordinated deformation mechanism [29]. Studies have also been done on multilayer maraging/CoCrNi composites, where the superior interface bonding helps with the composites’ toughening and deformation coordination [20]. Furthermore, an analytical–numerical method was proposed to determine the mechanical fields in the composite structures with interphase ribbon-like deformable multilayered inhomogeneities under combined force and dislocation loading. These studies focused on the deformation and strength parameters of composite structures with thin multilayer ribbon-like inclusions [28,30,31].

One key advantage of high-performance NMMCs is their exceptional strength-to-weight ratio. Due to their nanoscale structure and high strength, these composites can be used in various applications where lightweight materials with superior mechanical properties are required. For example, they have been explored for use in aerospace components, automotive parts, sporting goods, and protective coatings [32,33,34]. Furthermore, high-performance NMMCs exhibit excellent electrical conductivity due to their metallic nature. This makes them suitable for applications requiring high electrical conductivity combined with mechanical robustness. Examples include electrical contacts, interconnects in microelectronic devices, and electromagnetic shielding [3,12]. Another notable characteristic of high-performance NMMCs is their exceptional thermal stability. The presence of multiple interfaces within the composite structure acts as a barrier for heat transfer pathways [1,35]. This property makes them promising candidates for applications involving high-temperature environments or where efficient heat dissipation is crucial [1,35].

Despite their numerous advantages, there are challenges associated with high-performance NMMCs. One major challenge is achieving good adhesion between different layers during fabrication to prevent delamination or interfacial failure under mechanical stress [12,21,36]. Additionally, controlling the composition and thickness uniformity across large areas remains a technical hurdle [5,37]. With ongoing research efforts focused on improving fabrication techniques and addressing challenges related to scalability and reliability issues, these composites have a lot of potential for use in numerous industries and applications.

### 1.2. Motivation

Exploring the strengthening effects of grain size reduction, or the Hall–Petch effect, was the initial driving force behind the study of NMMCs [5,38]. These materials possess unusually high strength at thin layer thicknesses, making them attractive for various applications. NMMCs have been shown to have phonon-glass thermal conductivity, which makes them attractive for high-performance thermal barriers [7]. These materials can be used to make devices that require efficient heat management. Additionally, designing and synthesizing new materials with enhanced properties requires an understanding of the mechanical behavior of these materials. NMMCs have potential applications in various fields, including electronics, energy, and biomedical engineering. So, these materials can be used to make high-performance devices and structures. Studying NMMCs can lead to fundamental insights into the behavior of materials at the nanoscale [39]. This can help advance our understanding of materials science and eventually result in the creation of new materials with enhanced properties. Overall, studying high-performance NMMCs is motivated by the desire to understand and exploit the unique properties of these materials for various applications. These materials possess high strength, unique thermal conductivity, and tunable mechanical properties, making them attractive for various fields of research.

## 2. Synthesis Methods

The fabrication techniques for high-performance NMMCs involve various methods, such as physical vapor deposition, chemical vapor deposition, accumulative roll bonding (this method can be utilized for the successful fabrication of layered structures [25,40]), electrodeposition, and magnetron sputtering. These techniques allow precise control over layer thicknesses and material compositions to tailor the desired properties of the composite [41,42].

### 2.1. Physical Vapor Deposition

**Figure 3:**
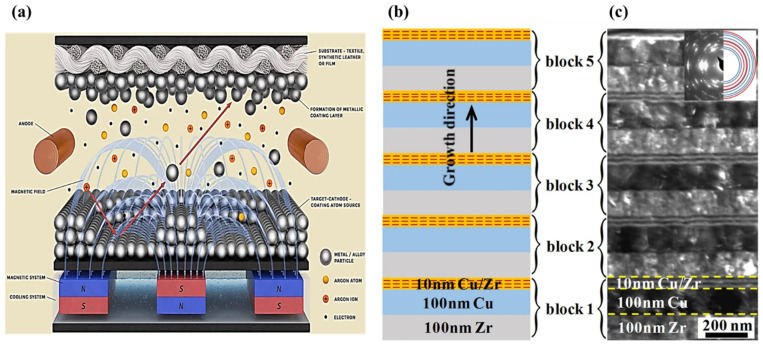
(**a**) the sputter deposition method as a physical vapor deposition (PVD) technique, (**b**) a schematic diagram of the hybrid Cu/Zr nanolayer architecture (HB_2_), and (**c**) dark field TEM image of the hybrid Cu/Zr nanolayer architecture, with an inset showing a chosen area diffraction pattern [51].

The production of high-performance nanoscale metallic multilayer composites is a common application for physical vapor deposition (PVD) techniques. Using physical processes like sputtering or evaporation, the material is transferred from a solid source to a substrate in the thin film deposition technique known as PVD [12,43]. In this regard, Figure 3a illustrates the sputter deposition method as a PVD technique that is utilized to deposit thin films onto a substrate by ejecting material from a “target” onto a “substrate” using the phenomenon of sputtering. In the context of nanoscale metallic multilayer composites, PVD methods are used to deposit alternating layers of different metals with nanoscale thicknesses. These multilayer composites exhibit unique properties, due to the interaction between the different layers at the nanoscale. There are several PVD methods commonly employed in the fabrication of these composites, including the following:

I. Thermal Evaporation: in this method, a solid metal source is heated to its evaporation temperature, and the resultant vapor condenses to create a thin film on a substrate. The substrate can be rotated or tilted during deposition to achieve uniform layer thicknesses [44];
II. Electron beam evaporation: similar to thermal evaporation, this method uses an electron beam to heat the metal source and create a vapor that condenses onto the substrate. Electron beam evaporation enables accurate control over deposition rates and enables the fabrication of complex multilayer structures [45,46];
III. Sputtering: sputtering is the process of ejecting atoms or molecules from a target surface by ionizing a target material with high energy. These ejected particles then deposit on a substrate placed in close proximity to the target [47]. Magnetron sputtering is commonly used for fabricating metallic multilayer composites, due to its high deposition rates and ability to control film composition [48], as shown in Figure 3a.
IV. Ion beam-assisted deposition (IBAD): IBAD combines ion beam bombardment with traditional PVD techniques. The ion beam assists in controlling film growth by enhancing adatom mobility on the substrate surface, resulting in improved film quality and reduced defects [49,50]. In one study, Li et al. [51] produced heterogeneous multi-nanolayer metallic architectures by means of magnetron sputtering. This hybrid multilayered material is composed of alternating Cu/Zr bilayers, 10 nm and 100 nm thick. Using the intrinsic strength, thickness, and strain hardening of the layers, it deforms compatibly under both stress and strain; this effect is known as synergetic deformation. Figure 3b,c shows the schematic architecture and microstructure of the hybrid Cu/Zr multi-nanolayer metallic architecture. Significant synergistic strengthening was induced by the achieved compatible deformation, i.e., with a total strength of 1.69 GPa, in comparison to the calculated results from the rule of mixtures (ROM), reaching 768 MPa (an 83% increase).

These PVD methods offer several advantages for fabricating nanoscale metallic multilayer composites. They allow for atomic scale control over layer thicknesses, composition, and microstructure. Additionally, PVD techniques can be easily scaled up for large-scale production and offer excellent adhesion between layers. Overall, physical vapor deposition methods play a crucial role in achieving high-performance NMMCs by enabling precise control over their structure and properties.

### 2.2. Electrodeposition

Electrodeposition methods are commonly used in the fabrication of high-performance NMMCs [52]. These composites consist of alternating layers of different metals or alloys, typically ranging from a few nanometers to a few micrometers in layer thickness. Several electrodeposition techniques can be employed to fabricate these composites, including pulse electrodeposition, direct current electrodeposition, and electrochemical co-deposition [53,54]. Each method has its advantages and can be tailored to achieve specific properties in the resulting composite [55].

**Figure 4:**
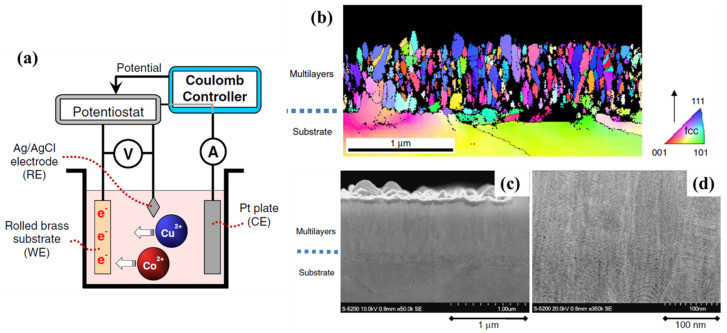
(**a**) Schematic of the electrodeposition device; (**b**) Co/Cu multilayer cross section inverse pole figure map. Cross-sectional Co/Cu multilayer FE-SEM images; (**c**) the electrodeposited sample’s entire cross section, in which the cross section preparation process most likely resulted in the contaminants covering the film surface; and (**d**) a larger image of the cross section of the film [59]. Note that the layer thickness is extremely close to the target value, and a zigzag multilayered structure is seen.

Pulse electrodeposition involves applying a series of current pulses with varying amplitudes and durations to the substrate. This technique allows for precise control over the deposition process, enabling the fabrication of multilayer composites with well-defined layer thicknesses and compositions. By adjusting the pulse parameters, it is possible to achieve different microstructural features, such as grain size, texture, and interface morphology [54]. Direct current electrodeposition is a simpler method, where a constant current is applied to deposit the metal layers onto the substrate. This technique is relatively easy to implement and can produce multilayer composites with good adhesion between layers. However, it may not offer as much control over layer thickness and composition as pulse electrodeposition [56,57]. Electrochemical co-deposition involves simultaneously depositing two or more metals in a single electrolyte solution. By controlling the deposition conditions, such as current density and bath composition, it is possible to achieve precise control over layer thicknesses and compositions in the resulting composite [58]. In one study, Takane et al. [59] utilized an electrodeposition method, as shown in Figure 4a, to fabricate Co/Cu multilayers. These Co/Cu multilayers were electrodeposited in a single electrolyte onto a brass substrate with a target layer thickness of 4 nm. As can be seen in Figure 4b,c, each sample consisted of vertically oriented columnar crystal grains with a multilayered Co/Cu structure in each grain. The layers formed a zigzag multilayered structure as a result of the layers being regularly bent at certain boundary lines in the cross section during the growth of the grains. This multilayered structure manifested significantly increased coercivity [59].

In all these electrodeposition methods, careful selection of electrolyte composition, deposition parameters (such as current density and deposition time), and substrate preparation are crucial for obtaining high-performance nanoscale metallic multilayer composites. When compared to their bulk counterparts, the resulting composites may have improved mechanical characteristics, like high strength, hardness, wear resistance, and improved thermal stability [60]. Overall, electrodeposition methods provide an effective means for fabricating high-performance NMMCs, with tailored properties for various applications in fields like electronics, energy storage devices, catalysis, and biomedical engineering.

### 2.3. Chemical Vapor Deposition

**Figure 5:**
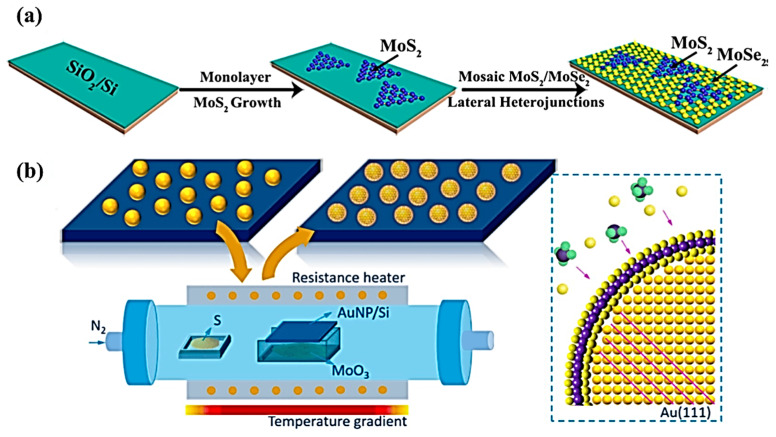
Chemical vapor deposition (CVD) techniques in the production of heterostructure and multilayers: (**a**) diagram illustrating the two-step CVD process used to create a film with mosaic MoS_2_-MoSe_2_ lateral heterojunctions on a SiO_2_/Si substrate [65] and (**b**) a schematic representation of the MoS_2_ shell growth process via CVD on Au nanoparticles [66].

One technique for creating high-performing nanoscale metallic multilayer composites is chemical vapor deposition (CVD). It involves the deposition of thin films of different metals onto a substrate through a chemical reaction in the vapor phase [61]. In CVD, a carrier gas and a precursor gas containing the desired metal atoms are introduced into a reaction chamber. The precursor gas can be in the form of metalorganic compounds or metal halides. The carrier gas helps transport the precursor gas to the substrate [62]. Inside the reaction chamber, the precursor gas undergoes thermal decomposition or reacts with other gases to form volatile metal compounds. These volatile compounds then come into contact with the heated substrate, where they decompose and deposit metallic atoms onto its surface. The deposition process can be controlled by adjusting various parameters, such as temperature, pressure, and flow rates of gases [63]. By carefully controlling these parameters, the multilayer composite’s layers can each have their thickness and composition precisely controlled. The CVD techniques can be successfully utilized to produce various types of multilayer structures and heterostructures, as demonstrated in Figure 5 [64]. For instance, Figure 5a demonstrates how Chen’s group [65] used a two-step CVD technique to produce a MoS_2_-MoSe_2_ lateral heterostructure film on a SiO_2_/Si substrate. Initially, SiO_2_/Si substrate was used to grow triangular MoS_2_ monolayers. Next, a continuous mosaic MoS_2_-MoSe_2_ lateral heterostructure membrane is created in the second CVD step by combining large-area MoSe_2_ film with the triangular MoS_2_ monolayers created in the first step. Furthermore, Li et al. [66] produced Au@MoS_2_ core-shell heterostructures by growing the multilayer MoS_2_ shell, resembling a fullerene, directly on the cores of Au nanoparticles. A MoS_2_ shell forms on the Au nanoparticles during deposition, as a result of a reaction between sulfur vapor and volatile MoO_3_ and its partially decomposed suboxides.

The NMMCs fabricated using CVD exhibit unique properties, due to their layered structure. The interfaces between different layers act as barriers for dislocation movement, resulting in improved mechanical strength and hardness, compared to bulk materials [67]. Additionally, CVD allows for precise control over layer thicknesses at the nanoscale level, enabling the tailoring of properties such as electrical conductivity and thermal stability [12,68]. Overall, CVD methods play a crucial role in fabricating high-performance NMMCs by providing precise control over layer thicknesses and compositions, leading to enhanced mechanical and functional properties.

### 2.4. Accumulative Roll Bonding

NMMCs can be produced using the accumulative roll bonding (ARB) process, which is a widely accepted method for fabricating multi-layered metal composites with microscale and nanoscale layer thickness [69,70,71]. Strong bonds are formed between the layers as a result of the material being repeatedly pressed and rolled during the ARB process [72]. Studies on nanostructured multilayers made by the ARB process, like Cu/Ni and Al/Cu/Mn, have been carried out on their mechanical characteristics and microstructural evolution [69,72]. Comparing ARB-produced NMMCs to conventional materials, these studies have shown that they have the potential to display superior mechanical properties like increased ductility and high strength [73,74].

**Figure 6:**
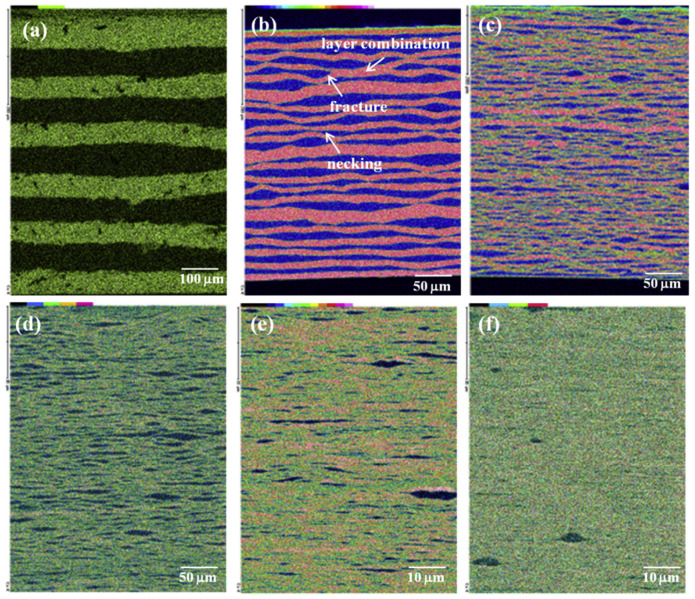
Cu elements in the multilayer after different ARB cycles: (**a**) 1, (**b**) 2, (**c**) 3, (**d**) 5, (**e**) 6, and (**f**) 7, which are mapped using SEM/EDS [69].

In an investigation by Sun et al. [69], ARB was used to fabricate a nanoscale Cu/Ni multilayer up to seven cycles by rolling pure Cu and Ni metal strips, leading to the overall composition of Cu_53_Ni_47_. Twin boundaries formed during the first four ARB cycles, and the Cu layer’s thickness, decreased slowly. The harder Ni layer is necked and fractured, resulting in the formation of the Ni island-like regions. Cu and Ni single-layer thicknesses homogenized after four ARB cycles; after seven ARB cycles, a single layer with a thickness of less than 100 nm was formed. In this regard, Figure 6 demonstrates the microstructure and distribution of Cu elements in the Cu/Ni multilayer after various ARB cycles. The Cu/Ni multilayer composite was found to have better mechanical properties than both Cu and Ni. For example, the multilayer’s ultimate tensile strength reached 950 MPa, which is roughly five times higher than the original pure Cu metal [69].

In another study by Watanabe et al. [71], the ARB method was utilized to produce nano-scale composite structures, and it was applied to thin Fe-Cu foils stacked alternately, which did not lead to solid solutions. It was revealed that to avoid layer fusion and separation during plastic deformation, the compression ratio for each pressing action needs to be less than 50%. Also, as the rolling reduction per pass increases, the layer structure gets smoother.

### 2.5. Atomic Layer Deposition

**Figure 7:**
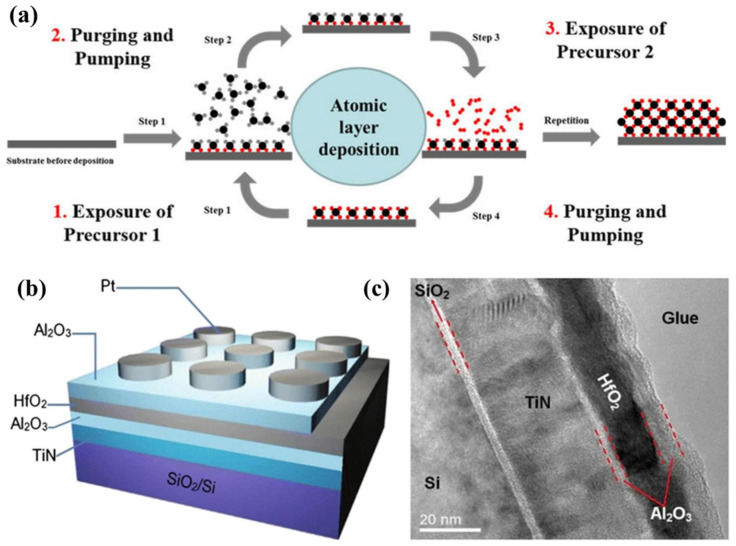
(**a**) a schematic description of the atomic layer deposition process [80], (**b**) a diagram and experimental setup of the ALD-produced TiN-coated Si Al_2_O_3_/HfO_2_/Al_2_O_3_ trilayer structure with Pt top electrode [79], and (**c**) a cross-sectional TEM image of the Al_2_O_3_/HfO_2_/Al_2_O_3_ trilayer structure on TiN-coated Si via ALD [79].

In order to create high-performance nanoscale metallic multilayer composites, a thin film deposition technique called atomic layer deposition (ALD) can be used. Thin films can be deposited with atomic-level accuracy using the highly controlled and precise ALD method. In ALD, the deposition process occurs in a cyclic manner, where there are two half-reactions in each cycle [75]. In the first half-reaction, the substrate is exposed to a precursor gas, which reacts with the surface to create an atomized monolayer. The second half-reaction involves the exposure to another precursor gas that reacts with the previously deposited monolayer, resulting in the growth of another atomic layer [76,77]; this process is schematically shown in Figure 7a. ALD allows for precise control of film thickness and composition, due to its sequential and self-limiting nature. It also enables conformal coating on complex three-dimensional structures, making it suitable for fabricating NMMCs [78]. In a study by Wang et al. [79], a trilayer structure of Al_2_O_3_/HfO_2_/Al_2_O_3_-based functional stacks on a TiN-coated Si substrate was fabricated by the ALD method, as shown in Figure 7b. A characteristic bipolar, reliable, and repeatable resistive switching behavior was displayed by this multilayer structure.

In the fabrication of high-performance nanoscale metallic multilayer composites, ALD can be used to deposit alternating layers of different metals or metal alloys. By controlling the thickness and composition of each layer, it is possible to tailor the properties of the composite material. ALD offers several advantages in fabricating these composites. Firstly, it provides excellent control over film thickness at an atomic level, ensuring uniformity throughout the material. Secondly, it allows for precise control over composition by selecting different precursor gases for each layer. This enables the tuning of mechanical, electrical, and thermal properties as desired. Furthermore, ALD can be used to deposit ultra-thin layers with high density and low defect density. This results in improved mechanical strength and enhanced performance, compared to conventional deposition methods [81,82]. Overall, atomic layer deposition methods play a crucial role in fabricating high-performance NMMCs by providing precise control over film thickness and composition, while maintaining excellent material quality.

### 2.6. Other Fabrication Techniques

There are several other techniques for the fabrication of high-performance nanoscale metallic multilayer composites, including spin and dip coatings, spray pyrolysis, electrophoretic deposition, laser metal deposition, and aerosol-assisted catalytic chemical vapor deposition (AACCVD). Spin and dip coatings are simple and low-cost methods for depositing thin films on substrates [83]. Spraying a precursor solution onto a heated substrate, a process known as “spray pyrolysis”, allows the solution to break down [83]. Electrophoretic deposition is a method that involves the deposition of charged particles onto a substrate under the influence of an electric field [84]. Laser metal deposition is a technique that involves the use of a laser to melt and deposit metal powders onto a substrate [84]. AACCVD is a CVD technique that involves the use of an aerosol to transport the precursor gases to the substrate, resulting in improved deposition efficiency and reduced waste [85].

## 3. Mechanical Properties

High-performance NMMCs have unique mechanical properties that differ from those of bulk materials, due to their structure being composed of two or more metals stacked in alternating layers, with each layer only being a few nanometers thick. The mechanical properties of these composites are influenced by their microstructure, dislocation motion, and interface structure [86]. Here are some of the mechanical properties of high-performance nanoscale metallic multilayer composites:

I. Yield strength and hardness: due to the additional solid solution strengthening contribution in the BCC layer, the hardness and yield strength of NMMCs are higher than those of pure element FCC/BCC multilayers [5];
II. Ductility: the ductility of NMMCs is generally lower than that of bulk materials [21];
III. Fracture toughness: the fracture toughness of NMMCs is length-scale dependent, and phase transformation can enhance toughening [21];
IV. Wear resistance: NMMCs have been shown to have excellent wear resistance [36,87];
V. Radiation-induced embrittlement: NMMs have been found to exhibit enhanced radiation damage resistance [88]
VI. Plasticity instability: NMMs can exhibit plasticity localization and shear banding behavior [89].

### 3.1. Strength and Hardness Enhancement

**Figure 8:**
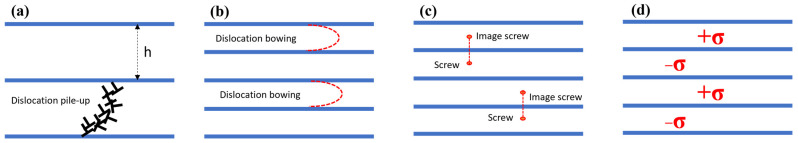
The schematic of possible strengthening mechanisms for metallic multilayers: (**a**) a model based on the Hall–Petch equation grounded on dislocation pile-ups at grain or interface boundaries (σ_ys_ ∝ h^−1/2^), (**b**) an Orowan model rooted on dislocation bowing between obstacles (σ_ys_ ∝ h^−1^lnh), (**c**) a Koehler model on the basis of image stress on dislocation in layer A (low shear modulus phase) from layer B (high shear modulus phase), and (**d**) alternating coherency stresses (±σ) between lattice-matched layers, leading to periodic resistance to dislocation motion across layers. Note that h shows the individual layer thickness in a multilayer where both layers have equal thicknesses, and σys is yield strength.

Strength and hardness enhancement are some of the key mechanical properties of high-performance NMMCs. There are some ways to enhance the strength and hardness of these composites. One way is to utilize solid solution strengthening. The higher hardness of nanoscale metallic multilayers, compared to pure element FCC/BCC multilayers, has been reported to be caused by the extra solid solution strengthening contribution in the BCC layer [90]. In this regard, Misra and Kung [90] claimed that metallic multilayers can possess ultra-high strengths under the condition that the bilayer periods range from a few nanometers to several. In this regard, Figure 8 shows the possible strengthening mechanisms of metallic multilayers. According to the Hall–Petch-based model depicted in Figure 8a, dislocations continue to accumulate at grain/interphase boundaries until the combined applied stress and stress concentration from the pile-up are greater than the barrier strength, and allow slip across the boundary. Because there are fewer dislocations in the pile-up when the grain size is smaller, there is less stress concentration. As a result, slip transmission across boundaries requires greater applied stress, which raises strength as the microstructural scale decreases. According to the equation of Hall–Petch, the yield strength (σ_ys_) is inversely proportional to the square root of the layer thickness (h), where σ_ys_ ∝ h^−1/2^. This connection arises from the fact that shrinking grain size raises the yield strength by increasing the amount of applied stress required to move a dislocation across a grain boundary [90]. In the Orowan-based model shown in Figure 8b, at small layer thickness, slip may occur by bowing of dislocations between interfaces, rather than pile-up formation. This results in a correlation between layer thickness and yield strength that follows the equation σ_ys_ ∝ h^−1^ln(h), according to the Orowan model. The Burgers vector of the shear dislocation loop, known as the Orowan loop, is parallel to the loop plane. The Orowan model accurately predicts precipitate hardening in cases where the precipitates are not shearable. The extra stress required for dislocations to bypass is thought to be caused by order strengthening in the case of shearable particles, though. The dislocation character, spatial distribution of precipitates, elastic anisotropy, stacking fault energy, coherency, formation of ledges at the precipitate/matrix interface due to dislocation passage, and formation of an antiphase boundary at the interface, all play a role in determining the applicability limit of the Orowan model. Recent research has demonstrated the potential of nanoscale precipitates to serve as long-lasting dislocation sources for improved ductility and high strength [91,92,93]. As opposed to the Koehler-based model depicted in Figure 8c, multilayers with a significant mismatch in the shear moduli of the layers are covered by dislocations. In such circumstances, slip may not be transmitted across layers until a dislocation in the lower modulus phase has sufficiently overcome sizable repulsive image stress from the higher modulus phase. This model is postulated for thin layers where Frank–Read sources may not operate and dislocation pile-ups do not form. According to the Koehler model, σ_ys_ is independent of h. Koehler demonstrated that dislocations would need to overcome the significant repulsive image stress if one of the constituent layers had a significantly lower shear modulus. This is due to the repulsive force that the higher modulus phase exerts on the dislocation in the lower modulus phase, which must be overcome before slip can happen [90,94,95]. In the coherency stresses model illustrated in Figure 8d, coherency stresses are considered stresses that arise from the lattice mismatch between layers in very thin lattice-matched multilayers. Between layers, these stresses alternate between compressive and tensile forces, which results in a cyclical resistance to dislocation motion. In order to account for the misfit between layers, interfaces are semi-coherent above the critical thickness for coherency loss and have dislocation arrays. Dislocations cannot cross the interface because of the stress field created by the misfit dislocation array [96]. Two coupled field quantities—the stress (or the elastic strain) and the displacement field—determine the elastic state of such a coherent multilayer. In a regular multilayer, however, when h exceeds a critical value, the interfaces are no longer coherent and the stresses are no longer periodic. This is not the case when the layers are very thin, where the stresses are independent of the multilayer wavelength h. Instead, dislocation arrays are used in the interfaces to account for the misfit between layers [97,98,99,100].

In addition to the mentioned mechanisms, other procedures can lead to the improvement of strength and hardness in multilayered structures, such as heterostructure strengthening, back stress strengthening, and heterogenous dislocation distribution strengthening. In heterostructure strengthening, to increase a structure’s overall strength and failure resistance, it is necessary to create a structure with numerous layers or materials, each with a unique set of attributes. A higher degree of strength and hardness can be achieved by the structure by combining the properties of various materials [101]. The back stress strengthening technique involves applying compressive stress to the back of a structure or material to improve its strength and stability. This can be achieved by using materials with different thermal expansion coefficients or by applying an external load to the back of the structure [101]. Heterogeneous dislocation distribution strengthening occurs when the dislocations in a multilayered structure are distributed heterogeneously, leading to a more efficient strengthening mechanism. The presence of dislocations can create localized stress fields, which can help to distribute the applied load more evenly and improve the overall strength of the structure [102].

Yang et al. [103] report notable strain hardening and back stress strengthening in gradient-structured interstitial-free (IF) steel. When geometrically necessary dislocations (GNDs) accumulate, long-range stress, known as back stress, is produced. The gradient in grain size of the gradient structure causes mechanical incompatibility. This creates a strain gradient, which GNDs must account for. In addition to increasing yield strength, back stress also dramatically improves strain hardening, which increases ductility. The occurrence of a back-strengthening effect was also reported by Jin et al. [104] in the AlCoCr_2_FeNi_2_ high-entropy alloy, which led to excellent mechanical properties with enhanced tensile strength.

**Figure 9:**
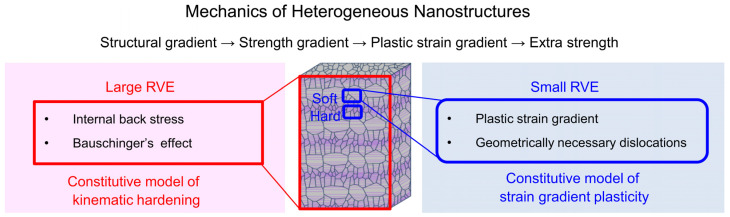
The mechanics of GNT Cu heterogeneous nanostructures regarding the selected representative volume element (RVE), relative to the characteristic length scales of GNT Cu [106].

The strengthening effect of heterogeneous dislocation distribution in metallic multilayers is a fundamental mechanism that enhances their mechanical properties. Increased strength and hardness are the result of the multilayered structure’s uneven dislocation distribution. Studies have indicated that ordered gradient nanotwinned (GNT) microstructures are highly controllable and can play a role in the basic mechanisms of strengthening metallic materials [105]. In this regard, Cheng et al. [106] investigate the source of the additional strength in gradient nanotwinned Cu, using a combination of controlled material processing, strain gradient plasticity modeling, back stress measurement, and dislocation microstructure characterization. The extra back stress resulting from the nanotwin thickness gradient is the main source of GNT Cu’s extra strength, whereas the effective stress is largely unaffected by the gradient structures. In this regard, Figure 9 demonstrates the mechanics of heterogeneous nanostructures in GNT Cu [106]. Furthermore, the presence of multielement elements and their different local chemical orders (LCOs) cause differentially active dislocations of the slip path and nanoscale segment detrapping processes to be triggered at the nano level, which governs the durability of the alloy [107]. Additionally, high dislocation density and heterogeneous nano/ultrafine particles have been shown to contribute to enhancing the strength and ductility of steel [108].

### 3.2. Ductility Improvement

**Figure 10:**
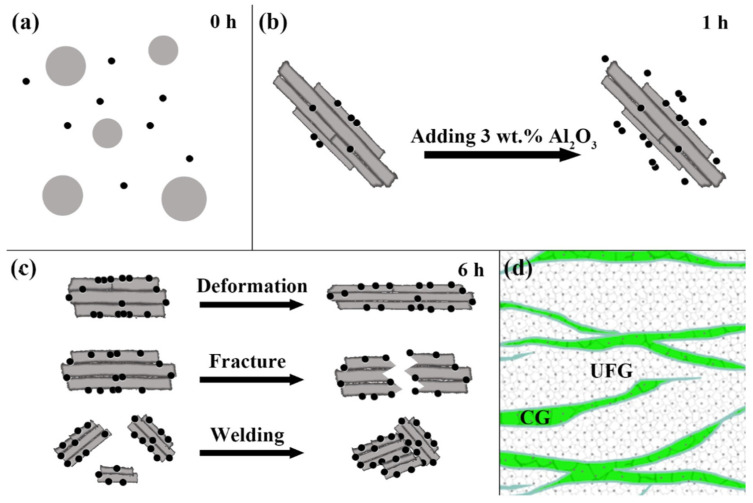
The creation of the heterogeneous lamella structure. The original Al powders (**a**) were deformed into a lamellar shape and cold welded after 1 h of high-energy ball milling at 200 rpm (**b**). The two-step addition of n-Al_2_O_3_ formed additional nanoparticle poor/free zones and the relatively short time of the milling process was only able to eliminate clusters by repeated deformation, fracture, and cold welding of Al powders (**c**). After hot forging, the flaky CG zones were stretched, resulting in a heterogeneous lamella structure (**d**). Note that green-colored locations represent nanoparticle-poor/free zones, i.e., coarse grain (CG) zones [110].

The numerous interfaces and thin nanoscale layer thicknesses of NMMCs have been shown to give rise to their exceptional properties [12]. However, low ductility has become a crucial challenge in nanoscale materials, including metallic multilayers [109]. A study conducted by Zan et al. [110] proposed a heterogeneous structure approach to achieve good synergy between strength and ductility for Al matrix composites. Their suggested heterogeneous lamella structure offered high elongation without compromising strength. The fabrication process of this heterogeneous structure is illustrated in Figure 10. In the beginning step, 2 wt.% n-Al_2_O_3_ particles were interspersed in the initial spherical Al powders. Following 1 h of high-energy ball milling at 200 rpm, the original Al powders (Figure 10a) were transformed and deformed into a lamellar shape and cold welded (Figure 10b), while the distribution of n-Al_2_O_3_ was still non-uniform and there was clear evidence of n-Al_2_O_3_ microclusters’ existence. There is a high possibility for cold welding in the region where the n-Al_2_O_3_ content is relatively low. Subsequently, with the addition of the rest of the n-Al_2_O_3_, they could only be scattered outside the welded powders, creating a difference in the amount of nanoparticle inside, compared to outside. As a result, the welded powders may produce nanoparticle-poor/free zones (NPZ) [111]. It was also claimed that the heterogeneous lamella composites with soft lamellar CG zones that were embedded in the ultrafine grains (UFGs) have considerably better strength and ductility performance over the other two conventional (bi-modal grain structure and the uniform UFG structure) structures [112].

Another study conducted by Fan et al. [113] demonstrated that coherent nano-lamellar alloys, which display an unprecedented ultra-high strength (over 2 GPa yield strength) and significant uniform tensile ductility, up to 16 percent, can achieve markedly enhanced tensile ductility. To achieve extremely high strength and ductility performance in Ni-Fe-Co-Cr-Al-Ti multicomponent alloys, they investigated a nanolamellar architecture using coherent L_12_ structures. The lamellar boundary strengthening was responsible for the extremely high strength of this composite, whereas the substantial ductility was brought on by a progressive work-hardening mechanism controlled by the special nano-lamellar architecture. It was claimed that the presence of coherent lamellar boundaries was the reason for facilitated dislocation transmission, removing boundary-related stress concentrations. Concurrently, unusually large tensile ductility results from networks of deformation-induced hierarchical stacking faults and associated high-density Lomer–Cottrell locks, which enhance the work hardening response [114]. In another study [21], a brief review of the mechanisms underlying cracking and toughening in nanoscale metallic multilayer films showed that the ductility and fracture toughness decrease with decreasing thickness below a certain threshold.

### 3.3. Fatigue Resistance and Fracture Toughness

NMMCs have unique properties, but their fatigue resistance and fracture toughness are still areas of research. The fracture toughness of metallic multilayer composites is influenced by interface slip resistance and strength, but limited decreases in overall strength have been observed [115]. According to a report, the primary factor influencing fracture and fatigue in thick, multilayer metallic systems is the resistance to slip transmission across the interphase boundaries [116]. Also, the indentation fatigue life is significantly influenced by hardness, and higher hardness is linked to longer fatigue lives [117].

**Figure 11:**
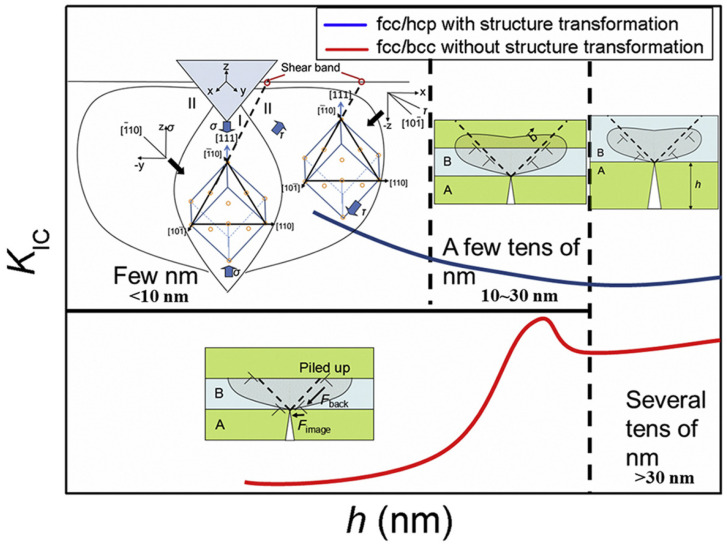
The schematic fracture toughness diagram shows systems that showed distinct mechanisms controlling cracking and fracture phenomena of the multilayer systems for both FCC/HCP multilayers, with and without lattice transformation. Note that the micro-mechanic fracture model schematics were also presented, emphasizing the interface crack shielding and slip system effects [119].

**Figure 12:**
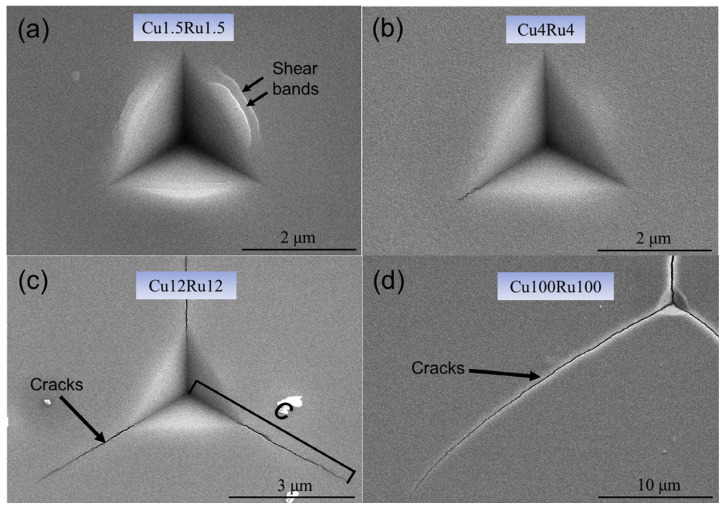
The SEM images of the morphologies of indentations in Cu/Ru multilayers with varied layer thicknesses: (**a**) Cu_1.5_Ru_1.5_, (**b**) Cu_4_Ru_4_, (**c**) Cu_12_Ru_12_, and (**d**) Cu_100_Ru_100_ multilayers [119].

Recently, Zauner et al. [118] proposed a novel method, using quasi-static and cyclic bending of pre-notched, unstrained microcantilever beams in conjunction with an in-situ synchrotron to study the fatigue resistance and fracture of nanostructured thin films. For example, Zhou et al. [119] optimized the layer thickness to increase the fracture toughness and crack resistance of Cu/Ru multilayer thin films. The films used to create the multilayered composites had equal individual layer thicknesses (h), ranging from 1.5 nm to 200 nm. It was found that as h was changed, a structure transition between the FCC and HCP lattices in the Ru layer occurred, affecting the Cu/Ru multilayers’ fracture toughness and length-scale-dependent cracking behavior. It is interesting to note that the Cu/Ru multilayer fracture toughness increased uniformly as the h values decreased to 1.5 nm. The main factors contributing to the improved toughness include the transformation of the lattice structure, reduction of h, and coupling effects from the increased interface transparency. In this regard, Figure 11 shows the fracture toughness schematic diagram for FCC/HCP multilayers, with and without lattice transformation. As can be seen, the multilayer of FCC/FCC Cu_1.5_Ru_1.5_ with the (111) out-of-plane texture should have numerous slip systems to support plastic deformation, as indicated in Figure 11’s left top corner. With compressive (region I) and shear (region II) stresses, Figure 12 depicts the slip condition of the Cu_1.5_Ru_1.5_ multilayer under nanoindentation. The (111) [110] slip systems of Cu_1.5_Ru_1.5_ could be triggered by further indenting of the indenter into the sample surface, which would result in the formation of shear bands, due to both compressive and shear stresses in the region I. Due to the limited number of slip systems present in the case of HCP Ru with (0002) out-of-plane texture, deformation under compressive stress is difficult to occur. In this regard, Figure 12 demonstrates SEM images of the indentation morphologies in Cu/Ru multilayers with different layer thicknesses [120]. As can be observed in Figure 12a, the remnant indentation of Cu_1.5_Ru_1.5_ only contained shear bands. In the Cu_4_Ru_4_ multilayer in Figure 12b, a triangle-shaped residual indentation has a clear indication of fabrication in the form of short cracks. Three mature radical cracks in Cu_12_Ru_12_ (Figure 12c) and Cu_100_Ru_100_ (Figure 12d) were revealed by a further h-increment. All Cu/Ru multilayers with different layer thicknesses were found to have much better ductile properties than other Cu/Ru multilayers, as the length of cracks decreased monotonically with decreasing h, until h = 1.5 nm without any crack formation around residual indentation [119].

### 3.4. Plasticity Instability

**Figure 13:**
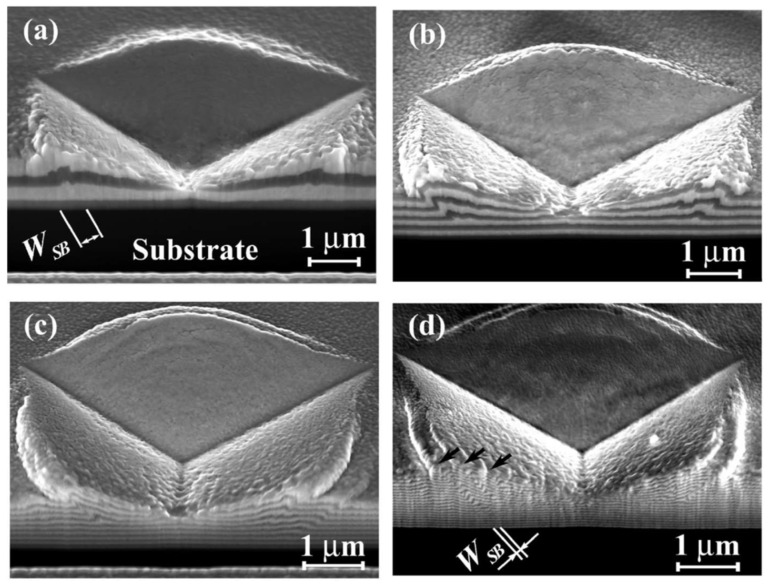
The focused ion beam (FIB) cross-sectional observation of the indents in the Au/Cu multilayers with varied layer thicknesses h: (**a**) 250 nm, (**b**) 100 nm, (**c**) 50 nm, and (**d**) 25 nm. Note that the bright and dark layers demonstrate the Au and Cu layers, respectively.

NMMCs are considered a class of materials that exhibit unique plasticity and stability characteristics [36]. However, NMMCs are prone to deformation-induced instabilities, such as strain localization, which can lead to crack nucleation and subsequent fracture [21]. According to experimental data, the multilayered composite can exhibit plastic deformation instability as the grain length scales and individual layer thicknesses get closer to the nanoscale. For instance, Zhang et al. [121] reported that, in the case of nanometric grain size/individual layer thicknesses, inhomogeneous shear banding becomes predominant in the multilayered Au/Cu composite. A substantial variation in shear banding behavior is shown in Figure 13 by the focused ion beam (FIB) cross-sectional observation of the indents in the Au/Cu multilayers with varying layer thicknesses, where the bright and dark layers represent Au and Cu layers, respectively. Figure 13 demonstrates unequivocally that as layer thicknesses are reduced, shear banding becomes more common. The plasticity and dislocation stockpile in small-scale crystals are significantly restrained at nanoscale grain size/layer thicknesses. Thus, with the aid of the very localized shear banding, inhomogeneous deformation combined with grain boundary sliding and grain rotation mechanism [121]. It has been proven that these multilayer NMMCs have limited deformability, as a result of localized deformation in the form of shear bands. The deformation mechanisms of NMMCs are length-scale-dependent, and the interface strengthening in NMMCs can lead to high strength [122]. However, there is ongoing research into the design of NMMCs for high strength, plasticity, and fracture resistance.

### 3.5. Radiation-Induced Embrittlement

Radiation-induced embrittlement in multilayer composites refers to the loss of ductility and the increased susceptibility to fracture due to radiation exposure. This phenomenon is attributed to various factors, including irradiation-induced defects such as dislocation loops, voids, and solute segregation, which can lead to the degradation of mechanical properties. Research has shown that radiation damage can result in amorphization, hardening, embrittlement, and swelling, ultimately causing the failure of the material [123]. Irradiation-induced debonding at the fiber/matrix interface has also been identified as a major cause of deterioration in various composites [124].

Radiation-induced embrittlement is a phenomenon that can occur in metallic nanolayered composites due to the formation of helium bubbles along grain boundaries [125]. The formation of these bubbles can lead to hardening, swelling, embrittlement, and surface deterioration, which can degrade the mechanical properties of the material [126]. However, metallic nanolayered composites have enhanced radiation resistance because of nanoscale repeat layer spacing m causing an exceptionally high density of heterophase interfaces [127]. The effective restraint of dislocation movements by the dense interface structure is what causes the strengthening mechanism in nanolayered structures. In some studies, the interfaces in Cu-V nanolayers prevented helium bubble aggregations, and it was determined that as repeat layer spacing was decreased from 50 nm to 2.5 nm, radiation hardening and swelling were significantly less severe in Cu-V nanolayers [127].

**Figure 14:**
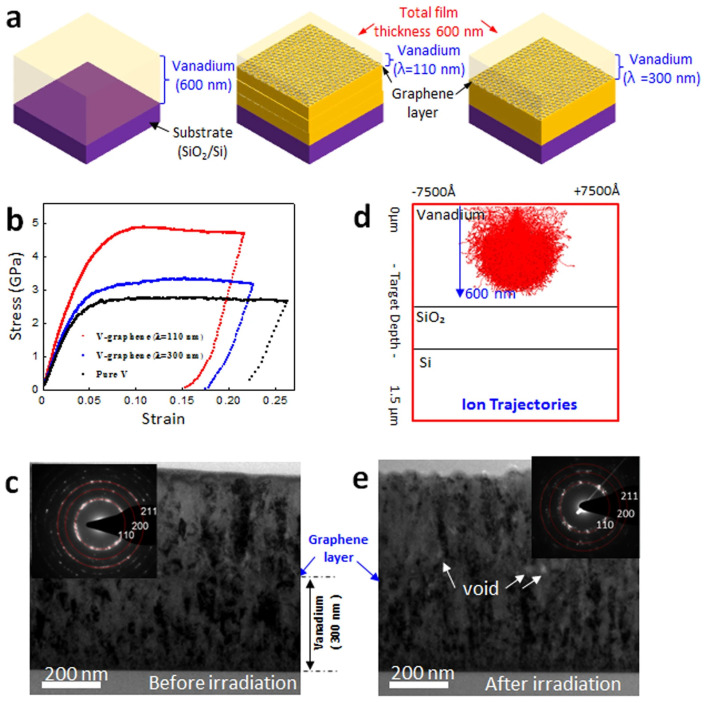
(**a**) Schematic for 110 nm and 300 nm repeat layer spacing (λ) in pure V and V-graphene nanolayers, (**b**) the stress-strain curve was derived from nanopillar compression testing of pure V and V-graphene nanolayers with repeated layer spacings of 110 nm and 300 nm, (**c**) TEM image demonstrating V-graphene’s nanocrystalline structure with repeated layer spacing of 300 nm, (**d**) He^+^ irradiation on V thin film at 120 keV with SRIM ion trajectories, and (**e**) TEM image demonstrating grain growth caused by radiation following He^+^ irradiation [127].

Figure 14a shows the V-graphene nanolayers with repeat layer spacings of 110 nm and 300 nm, along with a pure V thin film. As seen in Figure 14b, nanopillars were tested under compression after being synthesized from pure V. The average flow stress at five percent plastic strain was found to be 2.5 GPa, 3.1 GPa, and 4.8 GPa for pure V and V-graphene nanolayers with repeat layer spacings of 300 nm and 110 nm, respectively. It has been previously reported that the effective constraint on the dislocation motion across the interface is responsible for the strengthening effect of a single atomic layer thickness in graphene. Using a TEM, the microstructure of the synthesized V-graphene nanolayers with a 300 nm repeat layer spacing was examined. The V layers’ grain sizes are shown by both the micrograph and the selected area diffraction (SAD) to be in the tens of nanometer range (Figure 14c), which is substantially smaller than the grain sizes of Cu or Ni-graphene nanolayers that have previously been reported [128]. Also, SRIM (stopping and range of ions in the matter) ion trajectories of He^+^ irradiation on V thin film under 120 keV are shown in Figure 14d. In this regard, the radiation-induced grain growth after He^+^ irradiation was shown in the TEM images of Figure 14e. The research verified that the V-graphene nanolayers exhibited fewer radiation-induced crystalline defect formations, when compared to pure V. These findings aligned with the nanopillar compression results of the irradiated specimen V-graphene, which demonstrated a decrease in radiation-induced hardening and suppression of brittle failure [127].

## 4. Applications

### 4.1. Aerospace Industry

**Figure 15:**
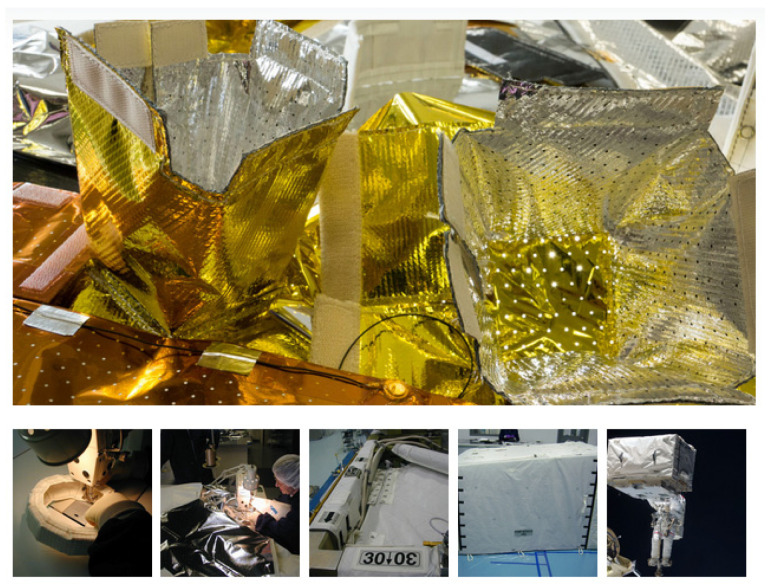
Multilayer insulation (MLI) blankets for passive thermal control in aerospace applications [130].

Numerous exceptional characteristics of NMMCs have been demonstrated, many of which are very different from those found in monolithic films. Due to their outstanding qualities, NMMCs offer a practical means of resolving the difficulties associated with developing and using new nanoscale engineering materials [12]. Nanocomposites have offered several different material solutions to the aerospace sector, including NMMCs, due to their advanced and immense mechanical properties. NMMCs’ application in the aerospace sector can potentially revolutionize the industry by offering a fuselage and structures that are lightweight and require minimal maintenance [129]. In this regard, as an example of the aerospace application of multilayer composites, they can be applied for a range of spacecraft, launch vehicles, and instruments operating in vacuum, multilayer insulation (MLI) blankets (such as plastics and metallic materials), where they offer passive thermal control. Through several layers of thin reflectors and spacer materials, they reduce the amount of radiative heat transfer, as shown in Figure 15 [130].

Some of the benefits of NMMCs in the aerospace industry include the following:

I. Enhanced mechanical properties, such as high yield strength and hardness;
II. High temperature and corrosion resistance [129];
III. Low grain growth in extremely hot conditions;
IV. Possibility of lightweight, low-maintenance structures and fuselage [129].

In summary, NMMCs have exceptional properties that make them a viable route to overcome the difficulties involved in creating and using new nanoscale engineering materials. In the aerospace industry, NMMCs can potentially revolutionize the industry by providing lightweight and low-maintenance fuselage and structures, enhanced mechanical properties, and high temperature and corrosion resistance.

### 4.2. Thermal Management Systems

The thermal conductivity of NMMs is an important property for their applications in various fields, such as thermal management in nanostructures, energy storage, and electronics. The thermal conductivity of NMMs is affected by the number of interfaces, the layer thicknesses, and the thermal conductivity of the individual materials. The thermal conductivity of NMMs is generally lower than that of the individual materials, due to the increased scattering of phonons at the interfaces [7]. For example, in the field of thermal management in nanostructures, the thermal conductivity of NMMs is critical for the design and optimization of thermal management strategies. The thermal conductivity of NMMs can be tailored by adjusting the number of interfaces, the layer thicknesses, and the thermal conductivity of the individual materials. This can help to improve the thermal conductivity of the NMMs and reduce the risk of thermal failure in nanostructures. Also, the thermal conductivity of NMMs can affect the performance of energy storage devices, such as batteries and supercapacitors. For example, a high thermal conductivity can help to improve the heat dissipation in energy storage devices, which can help to reduce the risk of thermal failure and improve the overall performance of the device [7].

**Figure 16:**
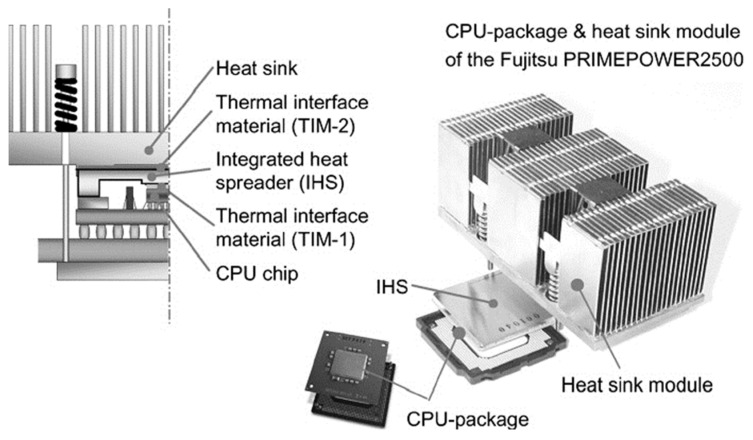
The design of the CPU system and heat sink module [135].

High-performance NMMCs can be used in thermal management systems for various applications, including the ones that follow:

I. Battery thermal management system: phase-changing composite material coupled to a metallic separator can be used for passive thermal design in large-format prismatic battery packs [131]. Another innovative idea is a dual-phase change material battery thermal management system based on petals [132];
II. Electronic devices: electronic equipment and powerful electrical systems are frequently cooled with heat sinks. Metal matrix composites can be used as reinforcements in heat sinks to improve their thermal conductivity [133]. Graphite and carbon nanofillers can also be used to improve the thermal performance of phase-change materials (PCMs) used in thermal management systems for electronic devices [134]. In this regard, Figure 16 shows the design of the CPU system and heat sink module [135]. The integrated heat spreader in this high-performance processor system is soldered or adhered to the chip using thermal interface material. A thermal interface material is used by the heat spreader to distribute heat from the chip to a larger area heat sink.
III. Satellite avionics: for satellite avionics and electronic components that are becoming more compact and powerful, thermal management systems are crucial. Metallic pin-fin geometries can be used to boost the thermal management performance of PCM-based modules [134]. Multilayer metallic composites at the nanoscale can also be used for thermal management in various systems and niche applications. Figure 16 shows the advanced thermal interface materials for high-power electronics applications with improved heat dissipation [136].

**Figure 17:**
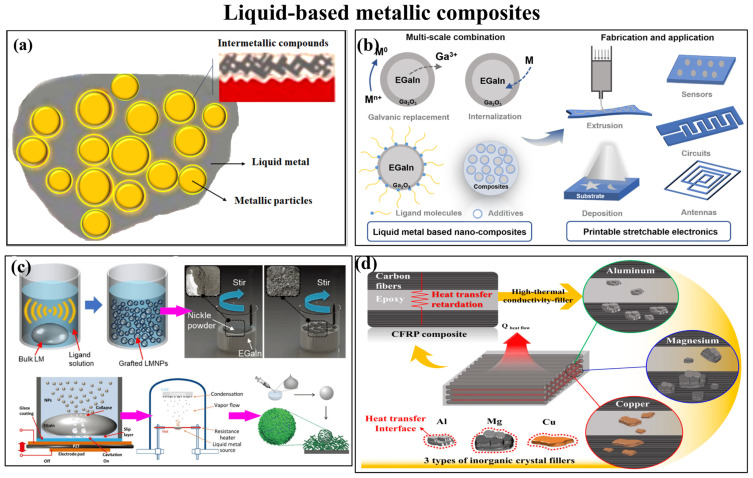
Some examples of liquid-based metallic composites: (**a**) the general structure of liquid-based metallic composites, (**b**) the nano-composites based on liquid metals and their use in printable stretchable electronics [142], (**c**) the basic synthesis routes for the production of liquid metal-based nano-composites [142], and (**d**) the layer-by-layer coated composites of high thermal conductivity carbon fiber-reinforced polymer and inorganic crystal fillers [143].

One of the examples of NMMCs in thermal applications is liquid-based metallic composites. Liquid-based metallic composites are materials that combine liquid metal with other materials, such as metal nanoparticles, polymers, and drug molecules [137]. These composites have recently attracted attention, particularly in thermal applications and flexible electronics applications, because of their distinctive characteristics [138]. Since it can remain liquid at room temperature or below, liquid metal (LM) has generated a lot of interest in thermal management [139]. Molecular thermal linkers have been used to create liquid metal composites with improved thermal conductivity and stability [140]. Also, liquid metal thermal interface materials (TIMs) outperformed traditional TIMs, in terms of performance, which are limited by their relatively low thermal conductivity [141]. High thermal and electrical conductivity, exceptional fluidity, and good biocompatibility have all been achieved in liquid metal composites, which have potential applications in thermal management. In this regard, Figure 17 shows some examples of liquid-based metallic composites; Figure 17a shows the general structure of these composites, Figure 17b demonstrates liquid metal-based nano-composites and their application in printable stretchable electronics, and Figure 17c shows the basic synthesis routes for the production of liquid metal-based nano-composites [142]. Additionally, Figure 17d demonstrates the use of a layer-by-layer coating to create carbon fiber-reinforced polymer composites with high thermal conductivity that contain inorganic crystal fillers [143]. Overall, liquid-based metallic composites have shown great potential in thermal management applications due to their unique properties, like high thermal conductivity, excellent fluidity, and biocompatibility.

### 4.3. Automotive Industry

**Figure 18:**
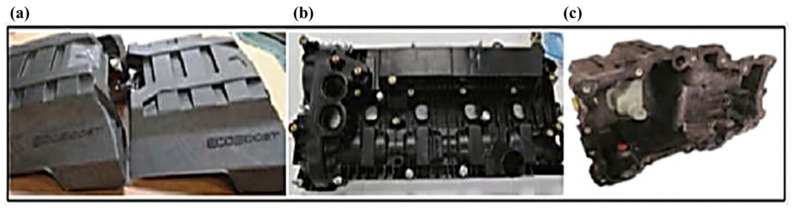
Some automobile components produced from metal matrix composites: (**a**) engine covers, (**b**) cam covers, and (**c**) oil pans [147].

NMMCs have shown great potential in the automotive industry, particularly in the development of cutting-edge tools and coatings for vehicles. By offering more wear protection and better heat dissipation, NMMC coatings can be specifically designed to increase tool durability and cutting efficiency [144]. These nanocoatings can be made to reduce friction as well, increasing productivity and reducing tool wear. Additionally, these coatings contain particular additive elements that support coating characteristics, like lubricant retention, corrosion resistance, and increased thermal stability [145]. NMMCs have been studied for their mechanical and fracture-related properties, including cracking and toughening mechanisms [21]. All these advantageous aspects lead to numerous applications in the automotive industry. For instance, nanomaterials have been investigated for use in the fatigue strength of aluminum-based metal matrix composites in multi-material vehicles with composite parts [146]. In this respect, Figure 18 shows some automobile components produced from metal matrix composites, including engine covers, cam covers, and oil pans [147].

### 4.4. Electronics Industry

The development of new materials and structures for electronic devices is one area where NMMCs have demonstrated great promise. Cu/Nb composites, for instance, can be made sustainably and have excellent electrical conductivity and ultrahigh strength due to their continuous laminated structure [148]. Here are some applications of NMMCs in the electronics industry:

I. Printable stretchable electronics: liquid metal-based nanocomposites have been developed for printable stretchable electronics, which have potential applications in wearable devices and soft robotics [142];
II. Embedded passives and interconnects: nanomaterials have been explored for use in embedded passives and interconnects, which can improve the performance and reliability of electronic devices [149];
III. Sensors: NMMCs have been explored for use in sensors, such as gas sensors and biosensors, owing to their special qualities, which include sensitivity and a large surface area [12];
IV. Transistors: NMMCs have been explored for use in transistors, which can improve the performance and efficiency of electronic devices [12];
V. Memory devices: NMMCs have been explored for use in memory devices, such as resistive random access memory (RRAM), which can improve the storage capacity and speed of electronic devices [12].

**Figure 19:**
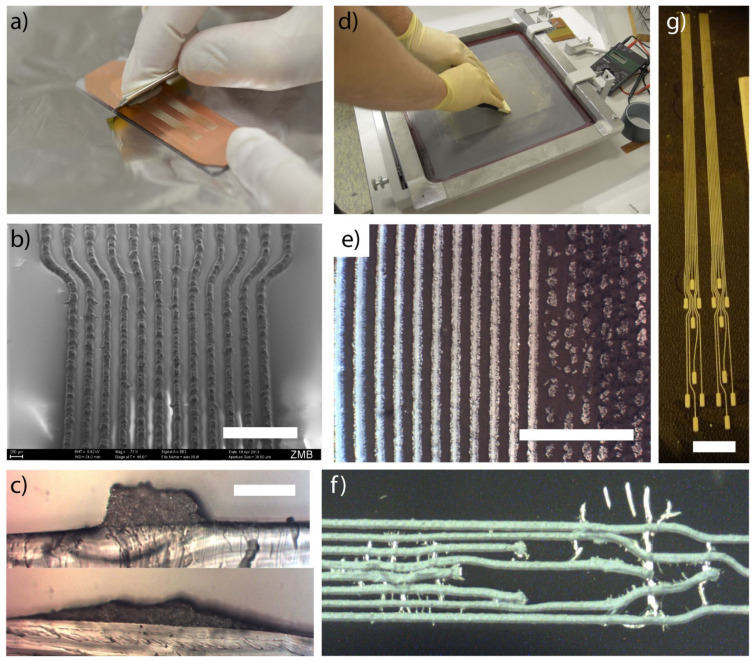
Creating soft PCBs using stencil and screen printing: (**a**) stencil printing of Ag-PDMS composites, (**b**) SEM micrograph of the stencil printed lines, (**c**) the cross section of stencil-printed Ag-PDMS (25 vol% (top) and 13 vol% (bottom)), (**d**) the screen-printing setup on 8” wafers, (**e**) a micrograph of the thinnest screen-printed lines (in black) and spaces (in white), (**f**) general flaws, like short circuits or PDMS layer separation from the glass substrate, and (**g**) very long screen-printed tracks. Note that scale bars are 0.1 mm in (**c**), 1 mm in (**b**,**e**), and 10 mm in (**g**) [150].

In one study, Larmagnac et al. [150] show the successful fabrication of a stretchable printed circuit board (PCB) from Ag-PDMS (poly(dimethylsiloxane) (PDMS)) composites, as shown in Figure 19. Their suggested route integrates main standard PCB design conditions such as connectivity, vias, straight traces, and solderability with standard hardware, and can be utilized to design soft and stretchable PCBs the same way rigid or flexible PCBs are designed. In another study, Deng et al. [149] fabricated SiO2-Pt nano-composite ceramic metal using the co-sputtering method, which can be used for embedded passives and interconnects. Also, graphene oxide inclusions coated with polydopamine and polydimethylsiloxane (PDMS) rubber have been incorporated into dielectric elastomer composites that can be used for embedded passives and interconnects [151]. A nano-layered Ni-P metallic glass composite coating with a compositionally modulated microstructure that additionally utilizes electrodeposition has been created that can be applied to embedded passives and interconnects [54]. Laterally embedded interconnects can also be developed using high-aspect-ratio polymer structures with electroless copper plating [152].

### 4.5. Energy Storage Devices

**Figure 20:**
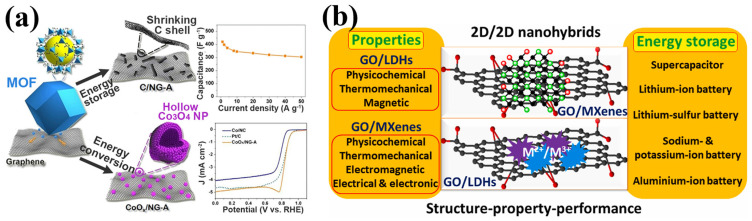
Some examples of energy storage applications of high-performance NMMCs: (**a**) metal oxide nanoparticles fabricated from bulk MOFs (Co-based MOF, Co(mIM)2 (mIM = 2-methylimidazole)) with supercapacitor applications [154]; and (**b**) 2D/2D nanocomposite based on graphene oxide-supported layered double hydroxides, and MXenes with numerous energy storage applications [153].

High-performance NMMCs have shown great potential for energy storage applications. Some of the possible applications include:

I. Supercapacitors: due to their excellent energy storage qualities, NMMCs are a promising material for supercapacitors [153,154,155];
II. Batteries: HPNMMCs can also be used in batteries, including lithium-ion, sodium-ion, and potassium-ion batteries [156];
III. Flexible energy storage devices: transition-metal chalcogenide nanostructures, including nanocrystals and thin films, are promising for flexible supercapacitors [156]. In this regard, Figure 20 shows some examples of energy storage applications of HPNMMCs, including (i) metal oxide nanoparticles fabricated from bulk metalorganic frameworks (MOFs) (Co-based MOF, Co(mIM)2 (mIM = 2-methylimidazole) with supercapacitor applications (Figure 20a) [154] and (ii) 2D/2D nanocomposite based on graphene oxide-supported layered double hydroxides and MXenes with numerous energy storage applications (Figure 20b) [153].

### 4.6. Biomedical Engineering

NMMCs have been used in various biomedical engineering applications. Due to their prevalence in both the human body and biomedical devices, metallic materials are among the most common materials used in biomedicine. These systems’ organized crystalline or amorphous structures, which are present at the nanoscale, have several unique properties that support useful biomedical applications [157]. Here are some of the applications of these composites in biomedical engineering:

I. Medical implants: metallic materials, including NMMCs, are used in the development of medical implants, due to their excellent physical and mechanical properties. These composites can be used to develop implants that are strong, durable, and biocompatible [157];
II. Drug delivery: metallic nanomaterials, including NMMCs, can be used for drug delivery in biomedical applications. These composites can be designed to release drugs in a controlled manner, which can improve the effectiveness of the treatment [158];
III. Biosensors: NMMCs can be used in the development of biosensors for the diagnosis of diseases [159]. These composites can be designed to detect specific biomolecules, which can help in the early detection of diseases;
IV. Therapeutics for radiotherapy: metallic nanomaterials, including NMMCs, can be used in the development of therapeutics for radiotherapy [158]. These composites can be designed to selectively target cancer cells, which can improve the effectiveness of the treatment;
V. Tissue engineering: NMMCs can be used to develop scaffolds for tissue engineering, as they can mimic the structure and mechanical properties of natural tissues [160].

**Figure 21:**
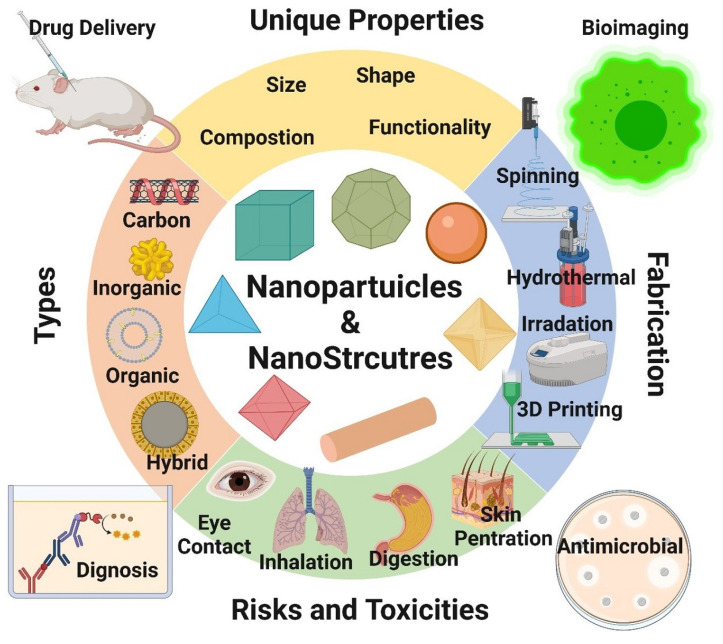
Some of the biomedical applications of nanoparticles, nanostructured materials, and nanoscale metallic multilayer composites [160].

In this regard, Figure 21 briefly introduces some of the biomedical applications of nanoparticles and nanostructured materials. Overall, NMMCs have great potential in biomedical engineering, and ongoing research is exploring their various applications in this field.

## 5. Conclusions and Future Perspectives

A comprehensive overview of the applications and mechanical properties of nanoscale metallic multilayer composites was analyzed and explained in the present study through an extensive review of existing literature and experimental studies, highlighting the potential of NMMCs in various fields. The paper began by introducing the concept of nanoscale metallic multilayer composites and their unique structural characteristics. Furthermore, this paper thoroughly examined the mechanical properties of nanoscale metallic multilayer composites. The key factors influencing the mechanical behavior of NMMCs, such as layer thickness, interface structure, composition, and processing techniques, were thoroughly discussed, presenting experimental results from various studies to support the findings. The study then delved into the wide range of applications where these composites have shown promise, including aerospace, automotive, electronics, and biomedical industries. The present study emphasized the exceptional mechanical properties exhibited by these composites that make them suitable for use in high-performance applications. The fact that nanoscale metallic multilayer composites have better mechanical properties than their bulk counterparts is one significant finding that is highlighted in this paper. These properties include enhanced strength, hardness, wear resistance, fatigue life, and thermal stability. These improvements were attributed to the unique microstructural features at the nanoscale level.

Moreover, this study sheds light on the underlying mechanisms responsible for these enhanced mechanical properties. It discusses concepts, such as the Hall–Petch strengthening effect, dislocation interactions at interfaces, grain boundary strengthening, and strain partitioning between layers. Understanding these mechanisms is crucial for optimizing the design and fabrication processes of nanoscale metallic multilayer composites. In summary, this study provides a comprehensive analysis of the applications and mechanical properties of nanoscale metallic multilayer composites. It highlights their potential in various industries and emphasizes their superior mechanical performance, compared to bulk materials. The findings presented in this paper contribute to advancing our understanding of these composites and pave the way for further research into optimizing their performance for specific applications.

### Future Perspectives

The field of high-performance nanoscale metallic multilayer composites is rapidly evolving, and several exciting future perspectives can be explored. In this section, this study discusses potential advancements in the applications and mechanical properties of NMMCs:

I. Enhanced Mechanical Properties: One of the key areas for future development is the improvement of mechanical properties in MMCs. Researchers can focus on optimizing the layer thickness, composition, and interface design, to achieve superior strength, hardness, and toughness. By tailoring these parameters at the nanoscale level, it is possible to create NMMCs with unprecedented mechanical properties;
II. Multifunctional applications: NMMCs have already found applications in various fields, such as aerospace, automotive, electronics, and energy sectors. However, there is still immense potential for exploring new multifunctional applications. For instance, researchers can investigate the integration of NMMCs into biomedical devices or wearable electronics to enhance their performance and durability;
III. Environmental sustainability: as the world moves towards a more sustainable future, it is crucial to consider the environmental impact of materials used in various industries. Future research can focus on developing environmentally friendly synthesis methods for NMMCs that minimize waste generation and energy consumption. Additionally, exploring the recyclability and reusability aspects of NMMCs will be essential for their long-term sustainability;
IV. Advanced manufacturing techniques: the development of advanced manufacturing techniques will play a significant role in realizing the full potential of MMCs. Additive manufacturing (3D printing) offers exciting possibilities for fabricating complex geometries with precise control over material composition and microstructure. Further advancements in this area can enable rapid prototyping and customization of NMMC components;
V. Computational modeling and simulation: with the increasing complexity of NMMC structures at the nanoscale level, computational modeling and simulation techniques will become indispensable tools for understanding their behavior under different loading conditions. Future research should focus on developing accurate models that can predict mechanical properties and failure mechanisms in NMMCs with high fidelity;
VI. Integration with other materials: combining NMMCs with other advanced materials, such as polymers and ceramics, can lead to synergistic effects and open up new avenues for applications. Future studies should explore hybrid material systems that leverage the unique properties of each constituent material to achieve enhanced performance across multiple domains
VII. Scale-up Challenges: while significant progress has been made in synthesizing nanoscale metallic multilayer composites at laboratory scales, scaling up production remains a challenge. Future research should address issues related to large-scale synthesis techniques while maintaining control over microstructure and mechanical properties.

In conclusion, high-performance NMMCs hold great promise for a wide range of applications, due to their exceptional mechanical properties. The future perspectives discussed above highlight some key areas where further research efforts can be directed to unlock their full potential and pave the way for innovative technologies across various industries.

## Data Availability

All data generated or analyzed during this study are included in this published article.
